# A Clinical Review of Continuous Glucose Monitoring in the Hospital Setting

**DOI:** 10.17925/EE.2025.21.2.3

**Published:** 2025-08-01

**Authors:** Jennifer N Clements, Kennedy Howard, Emory Moss

**Affiliations:** 1. Department of Clinical Pharmacy and Outcomes Sciences, University of South Carolina College of Pharmacy, Greenville, SC, USA; 2. University of South Carolina College of Pharmacy, Columbia, SC, USA

**Keywords:** Continuous glucose monitors, diabetes, diabetes technology, hospital, inpatient, monitoring

## Abstract

This editorial explores the critical role of continuous glucose monitoring (CGM) in managing diabetes within hospital settings. It provides a comprehensive clinical overview of CGM technology, highlighting its advances, such as improved glucose control and reduced hypoglycaemic events. This article also delves into the challenges, including cost and integration with existing hospital systems. The editorial examines real-world applications of CGM, highlighting its potential to enhance patient outcomes and streamline diabetes care in hospitals. By addressing both the current state and prospects of CGM, this article underscores its value in advancing diabetes management and improving overall patient care in hospital settings.

Approximately 20–34% of hospitalized patients have a diagnosis of diabetes, while many others experience stress hyperglycaemia, both increasing the occurrence of dysglycaemia in the hospital.^1^ Hyperglycaemia is associated with increased infection rates and higher mortality rates. Conversely, hypoglycaemia can lead to adverse neurological outcomes, prolonged hospital stays, as well as increased mortality.^1^ In spite of current guidelines and standards of practice, diabetes management can be challenging in the hospital due to a broad spectrum of hospitalized individuals with various glycaemic targets, diverse medications and individualized metabolic responses. There is a desire to have safe and effective non-pharmacological and pharmacological options for each individual and population as the landscape of diabetes management continues to evolve. Safe and effective diabetes management is especially important in the hospital to reduce mortality, improve quality of life and reduce the length of stay.^1^ Therefore, the use of continuous glucose monitoring/monitors (CGM) has emerged as a non-pharmacological and transformative diabetes technology to evaluate and manage glucose levels in hospitalized individuals.

CGM refers to a diabetes technology that allows for the continuous and real-time indirect measurement of glucose levels via an individual’s interstitial fluid.^2^ The technology is used to track and monitor glucose levels in real time throughout the day and night; a small sensor is inserted under the skin, typically in the abdomen or arm, to measure glucose levels in the interstitial fluid. The sensor transmits data to a monitor or smartphone app, providing continuous feedback on trends and patterns. This specific type of technology offers several advantages over traditional fingerstick testing with a glucometer. More data points from CGM provide a comprehensive view of glycaemic variability, allowing for more precise and timely adjustments, when needed, for improved glycaemic management.^2^ The comprehensive view can help detect hyperglycaemia and hypoglycaemia more effectively, reducing the risk of complications.

Initially instituted in the outpatient setting, where CGMs have become favoured and used, their potential for use in the hospital setting has emerged over recent years. Traditional glucometer testing methods are typically completed by staff four times a day in a non-critical, hospitalized patient, supplying limited snapshots of glycaemic status and increasing staff burden.^3,4^ With uninterrupted collection of data, CGM is a valuable tool for diabetes care in the hospital, as it can allow for timely and informed clinical decisions for hyperglycaemia and hypoglycaemia.^2^ Specifically focusing on insulin-dependent patients in the hospital, CGM innovations can monitor insulin dosing and administration to predict future hypoglycaemic or hyperglycaemic events using algorithms that anticipate the direction of blood glucose levels.^3^ This technological advancement provides improved accuracy and predictability of glycaemic events, making it attractive for inpatient use; however, the minimal research on CGM use in the hospital setting has limited its widespread implementation.^3,5^ With the increasing number of patients coming into the hospital with CGM devices, the need for additional research and guidance on in-patient use is needed.

This article provides a comprehensive exploration of the role of CGM as a tool for diabetes care in the hospital. The advantages and disadvantages of CGM are summarized with a perspective on clinical application to enhance care and improve outcomes in the dynamic environment of the hospital.

## Impact on outcomes

CGM is a powerful tool that can transform diabetes management in the hospital setting. Hyperglycaemia can increase the risk of infection, prolong hospitalization, delay wound healing and result in higher mortality rates.^1^ In the hospital, people living with diabetes or those with stress hyperglycaemia often experience fluctuating glucose levels due to stress, medications and food intake.^1^ Point-of-care testing with a hospital-approved glucometer may not fully capture glucose trends and could miss early signs of hyperglycaemia, hypoglycaemia or glucose variability. CGM offers significant benefits by providing continuous, real-time glucose data.^2^ This technology can detect hyperglycaemic or hypoglycaemic events earlier for timely intervention. Additionally, the frequency of fingerstick testing can be reduced, thereby decreasing the workload for bedside staff. CGM’s higher accuracy enables proactive management of diabetes care in the hospital.^1–3^

Several studies have highlighted the potential benefits of CGM in hospitalized patients, particularly in critical care settings.^6–12^ A randomized trial by Holzinger et al. demonstrated that real-time CGM can effectively monitor glucose levels in critically ill patients by reducing hypoglycaemic events without compromising safety.^6^ Similarly, a pilot study by Sweeney et al. found that CGM use in high-risk, non-critically ill post-cardiac surgery patients improved glycaemic control during intensive care unit transitions, especially in the COVID-19 era.^7^ Additional research suggested that hybrid CGM protocols reduced point-of-care glucose testing by 63%, achieved a mean time-in-range of 71.4% and significantly reduced recurrent hypoglycaemic events (p=0.03).^8^ Moreover, nursing acceptance of CGM is crucial for successful implementation, indicating a shift towards a broader adoption of diabetes technology in inpatient care.^9^ These findings underscore the need to redefine inpatient diabetes management strategies and support CGM integration into standard hospital protocols. *Table 1* summarizes articles published in the previous 12 months on CGM in the hospital, reflecting the growing popularity of CGMs for diabetes care in this setting and among diverse populations.^10–12^

## Considerations for implementation among hospitals

### Policy development and protocol standardization

The successful adoption of CGM in hospital settings requires careful planning and consideration by key stakeholders. Hospitals should establish comprehensive policies and protocols to ensure safe and effective implementation of this sophisticated diabetes technology. These should specify clear patient selection criteria, procedures for sensor insertion, calibration guidelines and alarm thresholds. Selecting appropriate patients who would benefit from CGM during hospitalization, including those with diabetes or stress hyperglycaemia, is critical. Following evidence-based guidelines and consensus reports ensures that CGM is used appropriately and consistently across hospital settings. Protocols should align with institutional practices and emphasize standardization to improve glycaemic management outcomes. The successful integration of CGM into protocols is a critical step towards improving diabetes care and management for hospitalized individuals.^3,5^

### Integration into clinical workflow

Hospitals should evaluate the best CGM device for implementation based on critical factors such as sensor accuracy, alarm functionality, data integration capabilities, ease of use and compatibility with electronic health records (EHRs). For patients with diabetes or stress hyperglycaemia, seamless integration of CGM data into the EHR is essential. This ensures real-time accessibility of glucose trends and alerts for the healthcare team and provider, enabling timely and informed clinical decision-making. Effective integration of CGM data into EHR systems is crucial for real-time monitoring and clinical decision-making. Seamless data access allows the healthcare team to recognize trends, mitigate risks like hypoglycaemia or hyperglycaemia and optimize treatment plans promptly. Additionally, hospitals must ensure compliance with privacy regulations, as well as adherence to other regulatory requirements.^3,5^

### Types of continuous glucose monitoring devices for hospital use

Available options include Food and Drug Administration (FDA)-approved invasive CGMs such as Dexcom G6/G7 (Dexcom Inc., San Diego, CA, USA), FreeStyle Libre systems (Abbott Laboratories, Abbott Park, IL, USA, Medtronic Guardian Sensor 3 (Medtronic, Galway, Ireland) and Eversense (Senseonics Holdings Inc., Germantown, MD, USA) are subcutaneous devices that are increasingly considered for hospital use. Among these, devices such as Dexcom G6 and Libre Freestyle Pro stand out due to their real-time data-sharing capabilities and minimal calibration requirements, making them promising candidates for inpatient care. Cost considerations, including sensor replacement, should be balanced with potential savings from improved glycaemic control and reduced complications. Selecting the right device for specific hospital needs maximizes the benefits of CGM technology. Healthcare providers should proactively order this technology, when appropriate, and be educated on the established criteria.^5^

### Education for healthcare teams and patients

For successful implementation, healthcare providers, including nurses, medical practitioners, advanced practice providers and pharmacists, should receive comprehensive, role-specific training on the selected CGM device annually. Training should be tailored to individual responsibilities. For instance, nurses should focus on sensor insertion, proper sensor placement and calibration techniques. In contrast, medical providers and pharmacists should concentrate on interpreting CGM data, recognizing glycaemic trends and making evidence-based therapeutic adjustments. This targeted approach ensures that all team members are equipped with the knowledge and skills necessary to seamlessly integrate and optimize CGM use to improve patient outcomes. Hospitalized individuals and family members should also be educated about the purpose and operation of CGM devices. Providing clear, accessible resources ensures confidence in device use and continuity of care after discharge.^5^

**Table 1:** Summary of recent articles on continuous glucose monitoring in hospital setting^10–12^

First author	Population	Intervention	Comparator	Primary outcome
Hirsch et al.^10^	People with type 2 diabetes, non-ICU	Glucose target 90–130 mg/dL guided by CGM	Glucose target 140–180 mg/dL	No significant improvement in glucose levels with CGM
Shang et al.^11^	People with COVID-19 in the ICU	Intermittent scanned CGM	Point-of-care capillary test	Significantly lower hazard ratio in 28-day mortality and shorter average ICU stay with the CGM group, compared to the control group
Oosterom-Eijmaelet al.^12^	People undergoing cardiac surgery	Real-time CGM	Point-of-care capillary test	Accurate method for glucose assessment despite intraoperative sensor interruptions

*CGM = continuous glucose monitoring; ICU = intensive care unit.*

### Continuous monitoring and outcome evaluation

Implementing CGM in hospitals is an ongoing process requiring regular assessment of clinical outcomes. Metrics such as glycaemic variability, hypoglycaemia and hyperglycaemia rates, length of stay and staff satisfaction should be monitored. Hospitals should use these insights to refine protocols and justify CGM adoption as a cost-effective strategy for improving patient outcomes. By addressing these considerations and selecting the most suitable CGM system, hospitals can effectively implement and harness the full potential of CGM to enhance glycaemic control, prevent complications and improve patient care within the hospital setting. The benefits of CGM use in hospitalized individuals are diverse and apply to populations that pose unique challenges, such as critically ill patients or paediatric patients.^3,5^

### Challenges and limitations

While CGM holds promise in enhancing diabetes management in the hospital, its adoption and use are not without challenges and limitations.

Some of the main challenges can include, but are not limited to cost, accuracy and calibration challenges, acceptance among hospital employees and data security and privacy concerns. Addressing these challenges and limitations is important to maximize the benefits of CGM implementation in a hospital setting. *Table 2* describes potential challenges and limitations with proposed solutions to overcome the barriers.

## Future directions

The future of CGM holds exciting possibilities for personalized diabetes management in the hospital; however, there may be more questions, indicating the need for ongoing clinical research and publication of best practices for successful implementation of CGM in a hospital setting. Ongoing clinical trials and research studies should validate the benefits of CGM in various populations, such as critically ill individuals or those on dialysis. In addition, research can refine best practices for protocol development, candidate selection and data interpretation of CGM in the hospital. For example, the integration of CGM data could foster seamless and comprehensive data sharing, which would be an integral part of the hospital or healthcare system’s digital infrastructure for diabetes care. Clinical impact could consistently be assessed based on a reduction in hospital days or length of stay, hypoglycaemic episodes and hyperglycaemic episodes. Other outcomes could include the impact on hospitalizations and cost savings on the healthcare system. As positive or valuable insights are available, evidence-based guidelines and consensus reports should be updated to reflect new results. Lastly, the role of artificial intelligence algorithms should be investigated to determine how CGM systems can provide more predictive analytics in the hospital setting.

**Table 2:** Challenges and limitations of continuous glucose monitors in the hospital with potential solutions

What is the challenge or limitation?	How is the challenge or limitation related to CGM?	How can the challenge or limitation be resolved to use CGM in the hospital?
Cost considerations	High initial investment and ongoing maintenance costs Financial constraints for hospitals, especially smaller or underfunded institutions	A cost–benefit analysis would assess long-term savings and improved outcomes with CGM Favourable reimbursement strategies could be negotiated with insurance providers
Accuracy and reliability	Inaccurate readings due to interferences (e.g. concomitant medications) Potential for device malfunctions or data transmission errors Unreliable glucose trends and readings	Thoroughly educate prescribers on interactions with medications Add alerts into the electronic health system to flag for interactions with CGM Establish a calibration process to ensure consistency with data
Training and education with integration	Need for extensive training for healthcare staff due to the large volume of data generated by CGM devices Time and resources required to ensure all staff are proficient in using CGM technology and interpreting data accurately to make informed clinical decisions into already existing EHR	Create a streamlined process for staff with educational handouts for hospitalized individuals Ongoing training and support can build knowledge, confidence and skills with CGM
Regulatory and ethical concerns	Compliance with healthcare regulations and standards	Address privacy and data security issues related to continuous monitoring
Patient compliance and comfort	Patient discomfort or resistance to wearing CGM devices	Ensure patient adherence to CGM protocols
Resistance to change and clinical Implementation	Potential resistance from healthcare providers accustomed to traditional glucose monitoring methods and may not be suitable for CGM	Overcome scepticism and promote adoption of CGM technology

*CGM = continuous glucose monitoring; EHR = electronic health records.*

## Conclusion

This article provides a comprehensive overview of the use of CGM in the hospital setting, when clinically appropriate for an individual with diabetes. Using CGM in the hospital setting can offer a proactive approach to address challenges such as hyperglycaemia, hypoglycaemia and glucose variability. In addition, CGM can enable healthcare professionals to intervene quickly with prescribed regimens and optimize control while minimizing risks associated with glucose instability. This technology can empower people with diabetes and clinicians to provide person-centred, team-based care with informed decisions and treatment plans for optimal care. While not approved by the FDA for hospital use, policies and procedures can be created and established for the safe use of CGMs in both non-critical care and critical care settings. Emphasizing accuracy and reliability and implementing best practices for quality assurance, hospitals can maintain the highest standards of patient care in the use of CGM. These measures ensure that CGMs effectively contribute to improved glycaemic outcomes within the hospital setting. Real-world data and research could further justify safety and efficacy of CGMs for hospital use.

## References

[1] American Diabetes Association Professional Practice Committee. (2025). 16. Diabetes Care in the Hospital: Standards of Care in Diabetes-2025.. Diabetes Care..

[2] American Diabetes Association Professional Practice Committee. (2025). 7. Diabetes Technology: Standards of Care in Diabetes-2025.. Diabetes Care..

[3] Shaw JLV, Bannuru RR, Beach L (2024). Consensus considerations and good practice points for use of continuous glucose monitoring systems in hospital settings.. Diabetes Care..

[4] Thabit H, Rubio J, Karuppan M (2024). Use of real-time continuous glucose monitoring in non-critical care insulin-treated inpatients under non-diabetes speciality teams in hospital: A pilot randomized controlled study.. Diabetes Obes Metab..

[5] Galindo RJ, Umpierrez GE, Rushakoff RJ (2020). Continuous glucose monitors and automated insulin dosing systems in the hospital consensus guideline.. J Diabetes Sci Technol..

[6] Holzinger U, Warszawska J, Kitzberger R (2010). Real-time continuous glucose monitoring in critically ill patients: A prospective randomized trial.. Diabetes Care..

[7] Sweeney AT, Pena S, Sandeep J (2022). Use of a continuous glucose monitoring system in high-risk hospitalized noncritically ill patients with diabetes after cardiac surgery and during their transition of care from the intensive care unit during COVID-19: A pilot study.. Endocr Pract..

[8] Davis GM, Faulds E, Walker T (2021). Remote continuous glucose monitoring with a computerized insulin infusion protocol for critically ill patients in a COVID-19 medical ICU: Proof of concept.. Diabetes Care..

[9] Faulds ER, Dungan KM, McNett M (2023). Nursing perspectives on the use of continuous glucose monitoring in the intensive care unit.. J Diabetes Sci Technol..

[10] Hirsch IB, Draznin B, Buse JB (2025). Results from a randomized trial of intensive glucose management using CGM versus usual care in hospitalized adults with type 2 diabetes: The TIGHT study.. Diabetes Care..

[11] Shang J, Yuan Z, Zhang Z (2025). Effectiveness of continuous glucose monitoring on short-term, in-hospital mortality among frail and critically ill patients with COVID-19: Randomized controlled trial.. J Med Internet Res..

[12] Oosterom-Eijmael MJP, Hermanides J, van Raalte DH (2024). Continuous glucose monitoring and the effect of liraglutide in cardiac surgery patients: A substudy of the randomized controlled GLOBE trial.. J Cardiothorac Vasc Anesth..

